# Preparation and characterization of biomimetic silk fibroin/chitosan composite nanofibers by electrospinning for osteoblasts culture

**DOI:** 10.1186/1556-276X-7-170

**Published:** 2012-03-06

**Authors:** Jyh-Ping Chen, Shih-Hsien Chen, Guo-Jyun Lai

**Affiliations:** 1 Department of Chemical and Materials Engineering, Chang Gung University, Kwei-Shan, Tao-Yuan, Taiwan, 333, Republic of China

**Keywords:** nanofibers, electrospinning, chitosan, silk fibroin, composite, osteoblastic cells

## Abstract

In this study, we have successfully fabricated electrospun bead-free silk fibroin [SF]/chitosan [CS] composite nanofibers [NFs] covering the whole range of CS content (0%, 25%, 50%, 75%, and 100%). SF/CS spinning solutions were prepared in a mixed solvent system of trifluoroacetic acid [TFA] and dichloromethane. The morphology of the NFs was observed by scanning electron microscope, and the average fiber diameter ranges from 215 to 478 nm. Confocal laser scanning microscopy confirms the uniform distribution of SF and CS within the composite NFs. To increase biocompatibility and preserve nanostructure when seeded with cells in culture medium, NFs were treated with an ethanol/ammonia aqueous solution to remove residual TFA and to change SF protein conformation. After the chemical treatment, SF/CS NFs could maintain the original structure for up to 54 days in culture medium. Properties of pristine and chemically treated SF/CS NFs were investigated by Fourier transform infrared spectroscopy [FT-IR], X-ray diffraction [XRD], and thermogravimetry/differential scanning calorimetry [TG/DSC]. Shift of absorption peaks in FT-IR spectra confirms the conformation change of SF from random coil to β-sheet by the action of ethanol, which is also consistent with the SF crystalline diffraction patterns measured by XRD. From TG/DSC analysis, the decomposition temperature peaks due to salt formation from TFA and protonated amines disappeared after chemical treatment, indicating complete removal of TFA by binding with ammonium ions during the treatment. This was also confirmed with the disappearance of F1s peak in X-ray photoelectron spectroscopy spectra and disappearance of TFA salt peaks in FT-IR spectra. The composite NFs could support the growth and osteogenic differentiation of human fetal osteoblastic [hFOB] cells, but each component in the composite NF shows distinct effect on cell behavior. SF promotes hFOB proliferation while CS enhances hFOB differentiation. The composite SF/CS NFs will be suitable for bone tissue engineering applications by choosing a suitable blend composition.

**PACS: **87.85.jf; 87.85.Rs; 68.37.Hk.

## Introduction

Many polymers, including synthetic or natural ones, have been electrospun into nanofibers with diameters ranging from tens of nanometers to a few micrometers. Because of their intriguing characteristics such as large surface area, high porosity, and biomimetic of the structure and function of the natural extracellular matrices [ECMs] of native tissue, electrospun polymeric nanofibers [NFs] have found great interest in tissue engineering as scaffolding materials [1-3]. Natural ECMs in the body are mainly composed of two types of extracellular polymers, proteoglycans and fibrous proteins with fiber diameters ranging from 50 to 150 nm, depending on the tissue type [4]. Specifically, ECMs in bone have many protein fibers (e.g., collagen fibers and elastin fibers) and proteoglycans consisting of a protein core covalently bound to long chains of sulfated glycosaminoglycan [GAG] disaccharides (e.g., chondroitin sulfate and hyaluronic acid). Fabricating biomimetic NFs intended for tissue engineering applications should therefore be considered not only from the structural characteristics of the NF but also from its composition, such as combining suitable ECM components like proteins and polysaccharides to fabricate composite NFs [5].

Silk emitted by the silkworm consists of two main proteins, sericin and silk fibroin [SF], fibroin being the structural center of the silk and sericin being the sticky material surrounding it. The SF protein mainly consists of the recurrent amino acid sequence (Gly-Ser-Gly-Ala-Gly-Ala)*_n_*. Among the biodegradable and biocompatible polymers, SF was extensively studied as one of the candidate materials for biomedical applications because it has several distinctive biological properties including good biocompatibility, biodegradability, and minimal inflammatory reaction [6]. Various methods have been proposed to process SF into different kinds of scaffolds such as films, gels, sponges, and non-woven mats for biomedical applications [7], including NFs by electrospinning [8,9]. The SF NFs have been used to culture with chondrocytes, osteoblasts, and mesenchymal stem cells and reported to enhance cell proliferation and attachment [10-12]. Chitosan [CS] is a GAG-like linear polysaccharide composed of glucosamine and *N*-acetyl glucosamine linked in a β(1→4) manner and is deacetylated from chitin, the second most abundant natural biopolymer in the world [13]. This natural biomaterial is found in the shells of crustaceans and is with antibacterial, biocompatible, and biodegradable properties [14]. It is widely used for biomedical applications, such as wound dressings, drug delivery carriers, and tissue engineering scaffolds [15]. The CS NFs have also been produced by electrospinning [16].

Composite NFs are promising as tissue engineering scaffolds by blending protein and polysaccharide in the spinning solution. However, a well-designed processing condition must be found beforehand to fabricate bead-free nano-sized fibers from the blend spinning solution during the electrospinning step. Previously, collagen/CS NFs have been prepared and used as wound dressings and tissue engineering scaffolds [17,18]. Both endothelial cells and smooth muscle cells proliferated well on these composite NFs. SF/chitin NFs were found to enhance keratinocytes and fibroblasts attachment and spreading [19]. SF/CS NFs have also been fabricated, but the maximum CS content in the composite can only reach 30 wt.% [20]. Application of this composite scaffold for bone tissue engineering applications will be promising as CS is beneficial for osteogenic differentiation only if the CS content can be raised.

In this study, electrospun SF/CS composite NFs covering the whole range of CS content from 0 to 100 wt.% were successfully fabricated using a trifluoroacetic acid [TFA]/dichloromethane [DCM] mixed solvent system. To make the NFs suitable for bone tissue engineering applications, a new chemical treatment method using ethanol/ammonia was developed to completely remove harmful TFA residues from the NFs and make the NFs insoluble in culture medium. The chemically treated NFs were shown to promote the proliferation and osteogenic differentiation of human fetal osteoblastic [hFOB] cells by the action of the SF and CS component in the NFs, respectively.

## Methods

### Preparation of SF/CS blend solution

*Bombyx mori *silk fibers were treated twice with 0.5% (*w/w*) NaHCO_3 _solution at 70°C for 30 min and then rinsed with 70°C distilled water to remove sericin. Degummed silk was dissolved in a mix solvent system of CaCl_2_/CH_3_CH_2_OH/H_2_O (mole ratio, 1:2:8) at 70°C for 6 h and filtered to get a SF solution. After dialysis in a cellulose dialysis tubing (MWCO = 50,000) against distilled water for 5 days with water change every 12 h, the SF solution was lyophilized to obtain regenerated SF sponges. Chitosan (MW = 1 × 10^5^, degree of deacetylation = 98%) was purchased from Fluka (Buchs, Switzerland). CS and SF solutions were prepared in a mixed solvent system of TFA/DCM (weight ratio = 7:3) at concentrations of 8 and 12.5 wt.%, respectively. CS/SF blend solutions with different SF/CS weight ratio (75:25, 50:50, and 25:75) were prepared in the same solvent system at 12, 10, and 9 wt.% (combined weight of CS and SF), respectively.

### Preparation of SF/CS nanofibers by electrospinning and chemical treatment

The system for electrospinning includes a glass syringe, a 22-gauge stainless-steel needle, a syringe pump (KD Scientific Co., Holliston, MA, USA), a high-voltage power supply (Glassman, High Bridge, NJ, USA), and an aluminum foil as the collector [21]. The distance between the needle tip and the collector was 16 cm. The syringe was mounted on the syringe pump, and spinning solution was drawn horizontally from the needle tip with an electrostatic force generated from the high voltage applied between the tip and the grounded collector. The applied voltage and flow rate were controlled at 18 kV and 0.3 ml/h, respectively. For chemical treatment, the SF/CS composite NFs were neutralized, crystallized, and insolubilized by immersing in 7% (*v/v*) ammonia solution/75% (*v/v*) ethanol aqueous solution for 30 min at room temperature.

### Measurement and characterization

The morphology of the NFs was observed by a scanning electron microscope [SEM] (Hitachi S3000N, Hitachi, Ltd., Chiyoda, Tokyo, Japan). The diameters were calculated by measuring at least 100 fibers from 10 images at random using ImageJ. Chemical analysis was carried out with Fourier-transform infrared spectroscopy using a Horiba FT-730 spectrometer (Horiba, Ltd., Kyoto, Japan) over a wavenumber range between 600 and 2,000 cm^-1 ^with a resolution of 2 cm^-1^. X-ray photoelectron spectroscopy [XPS] was performed with a Physical Electronics PHI 1600 ESCA spectrometer (Physical Electronics, Inc., Chanhassen, MN, USA) equipped with a spherical capacitor analyzer and a multi-channel detector. The X-ray source was generated with a magnesium anode at 15 kV and 400 W. The pressure in the analysis chamber was set below 2 ×10^-6 ^Pa. X-ray diffraction [XRD] patterns were recorded on a Siemens D5005 X-ray diffractometer (Bruker AXS, Karlsruhe, Germany) composed of a CuK_α _source, a quartz monochrometer, and a geniometric plate at a scanning speed of 2° min^-1 ^from 10° to 55°. Thermogravimetry/differential scanning calorimetry [TG/DSC] analysis was conducted with a Netzsch STA 449F1 (Netzsch Instruments, Inc., Burlington, MA, USA) from 25°C to 800°C at 10°C/min. Pore size of the nanofibrous membrane was measured by capillary flow porometry (PMI CFP-1100-AI, Porous Materials, Inc., Ithaca, NY, USA) using a wetting agent of 21 dynes/cm surface tension from four samples with three independent measurements for each sample.

Fluorescein isothiocyanate [FITC]-labeled chitosan (FITC-CS) and rhodamine [Rd]-labeled silk fibroin (Rd-SF) were used to prepare electrospun NF under the same processing condition to detect the presence of each component in the NF [17]. The image of FITC-CS and Rd-SF was obtained from confocal laser scanning microscopy (Zeiss LSM 510, Carl Zeiss AG, Oberkochen, Germany) where FITC-CS fluoresces green and Rd-SF fluoresces red. The excitation wavelengths for FITC and rhodamine are 495 and 54 nm, respectively. The emission wavelengths for FITC and rhodamine are 525 and 576 nm, respectively.

### Cell culture and analysis

Nanofibrous membranes were cut into disk shapes with 1.4-cm diameter, sterilized with 75% ethanol overnight, and rinsed three times with phosphate buffer saline before placing in 24-well culture plates (Nunc, Rochester, NY, USA). Human fetal osteoblastic cells (ATCC CRL-11372) were obtained from the American Type Culture Collection (Arlington, VA, USA), and cells at passage numbers 4 to 6 were used. An aliquot of 0.1 ml hFOB cells (4 × 10^5 ^cells/ml) was seeded onto the surface of the pre-wetted membrane in each well. Cell seeded scaffolds were incubated at 37°C for 4 h to allow cell adhesion, and the membrane was transferred to a new well with the addition of 1.5-ml culture medium (Dulbecco's modified Eagle medium [DMEM]/F12 (1:1) supplemented with 10% (*v/v*) fetal bovine serum, 1% (*v/v*) antibiotic-antimyotic) into each well. Cell culture was carried out at 37°C in a humidified 5% CO_2 _incubator with medium change every 3 days.

The viable cell number of hFOB at 0, 7, 14, 21, and 28 days was determined by (3-(4,5-dimethylthiazol-2-yl)-5-(3-carboxymethoxyphenyl)-2-(4-sulfophenyl)-2H-tetrazolium) [MTS] assays using the CellTiter 96 AQueous One Solution kit from Promega (Promega Life Sciences, Madison, WI, USA). The kit contains a novel tetrazolium salt which interacts with metabolically active cells to produce a soluble formosan dye. Colorimetric measurement of the formazan product was performed at 492 nm using an enzyme-linked immunosorbent assay [ELISA] plate reader (BioTek Synergy HT, Winooski, VT, USA). Alkaline phosphatase [ALP] activity of hFOB was measured using alkaline phosphate yellow liquid substrate system for ELISA (Sigma-Aldrich Corporation, St. Louis, MO, USA). Nanofibrous membranes with cells were washed with phosphate buffer saline and immersed in the lysis buffer. After centrifugation, the supernatant was collected and reacted with the substrate p-nitrophenyl phosphate for 30 min, and optical density of the colored product was measured with an ELISA reader at 405 nm. Cell growth and ALP activity were determined from five samples with four independent measurements for each sample.

Cell viability was also assessed by LIVE/DEAD Viability/Cytotoxicity Assay kit (Invitrogen, Carlsbad, CA, USA) which provides two molecular probes, calcein AM and ethidium homodimer-1 [EthD-1], to simultaneously determine the existence of live (green) and dead (red) cells. Cells were stained with 2 mM EthD-1 and 4 mM calcein AM and imaged under a confocal laser scanning microscope (Zeiss LSM 510). The excitation wavelengths for calcein AM and EthD-1 are 494 and 528 nm, respectively. The emission wavelengths for calcein AM and EthD-1 are 517 and 617 nm, respectively. For SEM observations, cell/scaffold samples were fixed in 3% glutaraldehyde, dehydrated through a graded series of ethanol soaks, and dried in a critical point dryer (Balzer CPD 030, Bal-Tec AG, Liechtenstein, Germany).

### Statistical analysis

All quantitative data were expressed as mean ± standard deviation. Statistical analysis was performed using the one-way ANOVA LSD test to determine significant differences. A value of *p *< 0.05 was considered statistically significant.

## Results and discussion

### Preparation of electrospun SF/CS nanofibers

**Figure 1:**
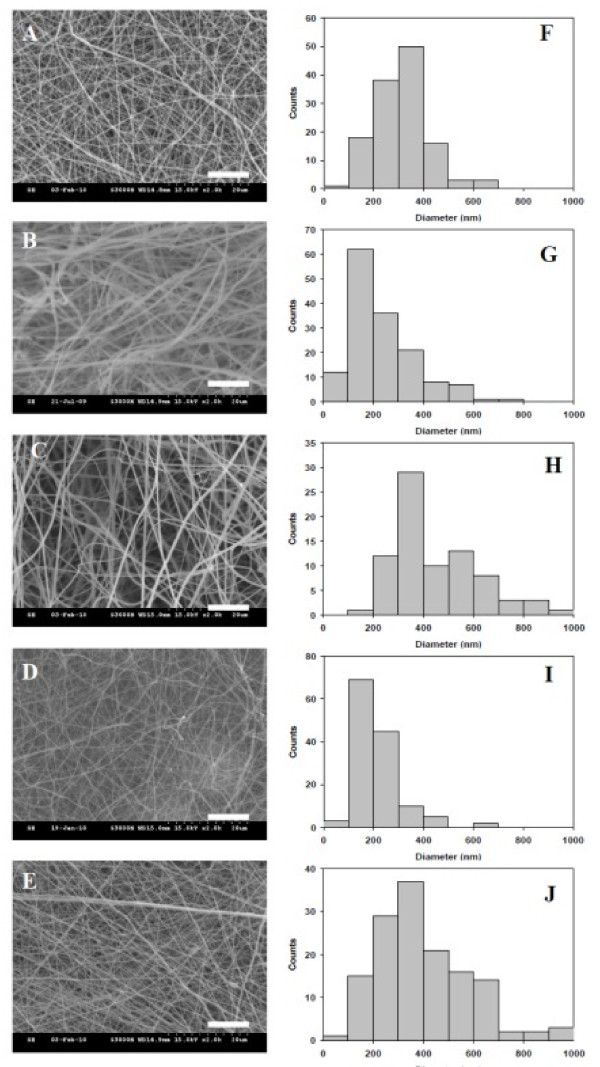
**SEM micrographs (A to E) and histograms of fiber diameters (F to J)**. Electrospun SF/CS nanofibers with different CS contents: (**A**), (**F**) 100% CS; (**B**), (**G**) 75% CS; (**C**), (**H**) 50% CS; (**D**), (**I**) 25% CS; and (**E**), (**J**) 0% CS. Bar = 10 μm.

**Figure 2:**
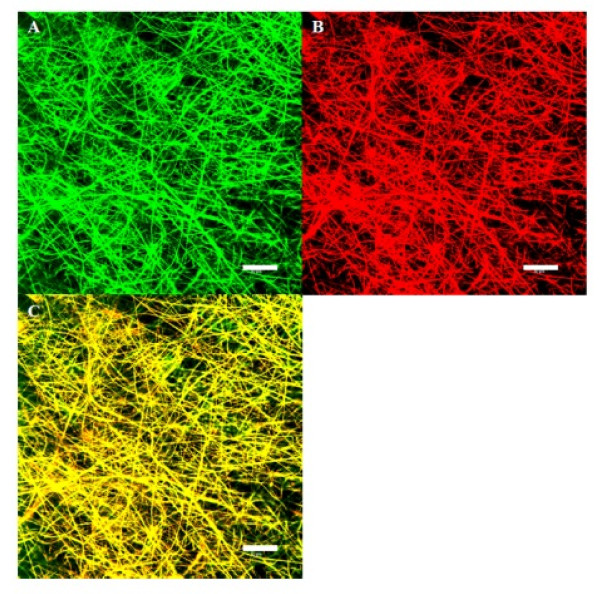
**Confocal laser scanning microscopy images of 50% SF/50% CS nanofibers**. (**A**) Distribution of CS by detecting FITC-CS, (**B**) distribution of SF by detecting Rd-SF, and (**C**) mergence of images (A) and (B). Bar = 50 μm. FITC fluoresces green and Rd fluoresces red.

As shown from SEM micrographs in Figure 1, bead-free and continuous SF/CS NFs with different CS contents could be prepared by choosing a suitable electrospinning conditions. The NFs are with a narrow fiber diameter distribution, and the average fiber diameter was calculated from Figure 1 and summarized in Table 1. The average fiber diameter ranges from 215 to 478 nm. The pore sizes of SF/CS membranes determined by capillary flow porometry are also included in Table 1 and range from 0.52 to 1.23 μm. To distinguish and visualize individual biopolymer component in the NF, FITC-CS and Rd-SF were used to prepare the electrospun 50% SF/50% CS NFs under the same fabrication conditions. The confocal microscopy images in Figure 2 confirm that the NF is composed of SF and CS, and the distribution of each constituent in the NF is uniform.

**Table 1:** Fiber diameters and pore sizes of SF/CS nanofibrous membranes

Composition of nanofibers	Fiber diameter (nm)	Mode (nm)	Q1 (nm)	Q3 (nm)	Pore size (μm)
SF	399 ± 184	143	271	516	0.98 ± 0.21
75% SF/25% CS	215 ± 95	141	152	253	1.23 ± 0.39
50% SF/50% CS	447 ± 168	340	340	544	0.71 ± 0.18
25% SF/75% CS	239 ± 133	143	143	309	0.73 ± 0.11
CS	317 ± 109	238	238	376	0.52 ± 0.10

Mode, first (Q1) and third (Q3) quartiles of fiber diameter.

**Figure 3:**
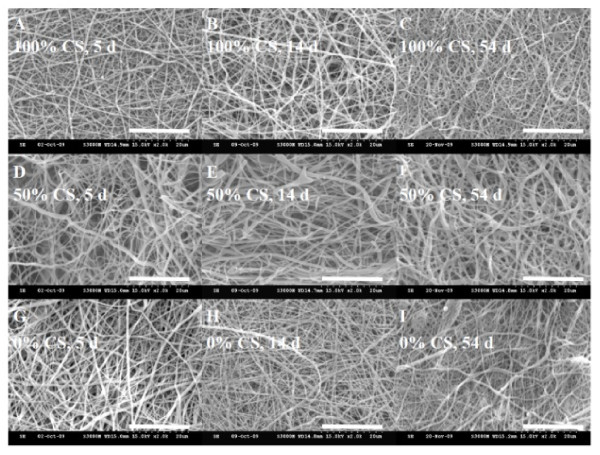
**Scanning electron micrographs**. Scanning electron micrographs of ammonia/ethanol treated SF/CS nanofibers with different CS contents after being immersed in cell culture medium for 5, 14, and 54 days. Bar = 20 μm.

As TFA will form salts with protonated amino groups of CS or SF, the NF could dissolve in aqueous solutions, which limit its use in cell culture and as tissue engineering scaffold. Residual TFA in the NF is also highly toxic towards cells and may lead to cell death if not removed completely. Chemical treatment of pristine NFs was therefore necessary. In this study, we have developed a new chemical treatment method using aqueous solution of ammonia and ethanol to serve the dual purpose of insolubilizing NFs and removing TFA simultaneously. TFA was removed by deprotonation of the amino groups by treatment in the alkaline solution (7% ammonia), which also made CS insoluble [22-24]. The SF was made insoluble by treating with the same aqueous solution containing 75% ethanol, which changed the conformation of SF from random coil to β-sheet [25]. Figure 3 showed the SEM micrographs of chemically treated NFs after being immersed in cell culture medium (DMEM/F-12 medium containing 10% FBS) for 5, 14, and 54 days, respectively. The SEM micrographs indicate electrospun NFs treated with 7% ammonia/75% ethanol aqueous solution could maintain their nanofibrous structure for a prolonged time period commonly encountered during cell culture.

### Analysis of SF/CS nanofibrous membranes

**Figure 4:**
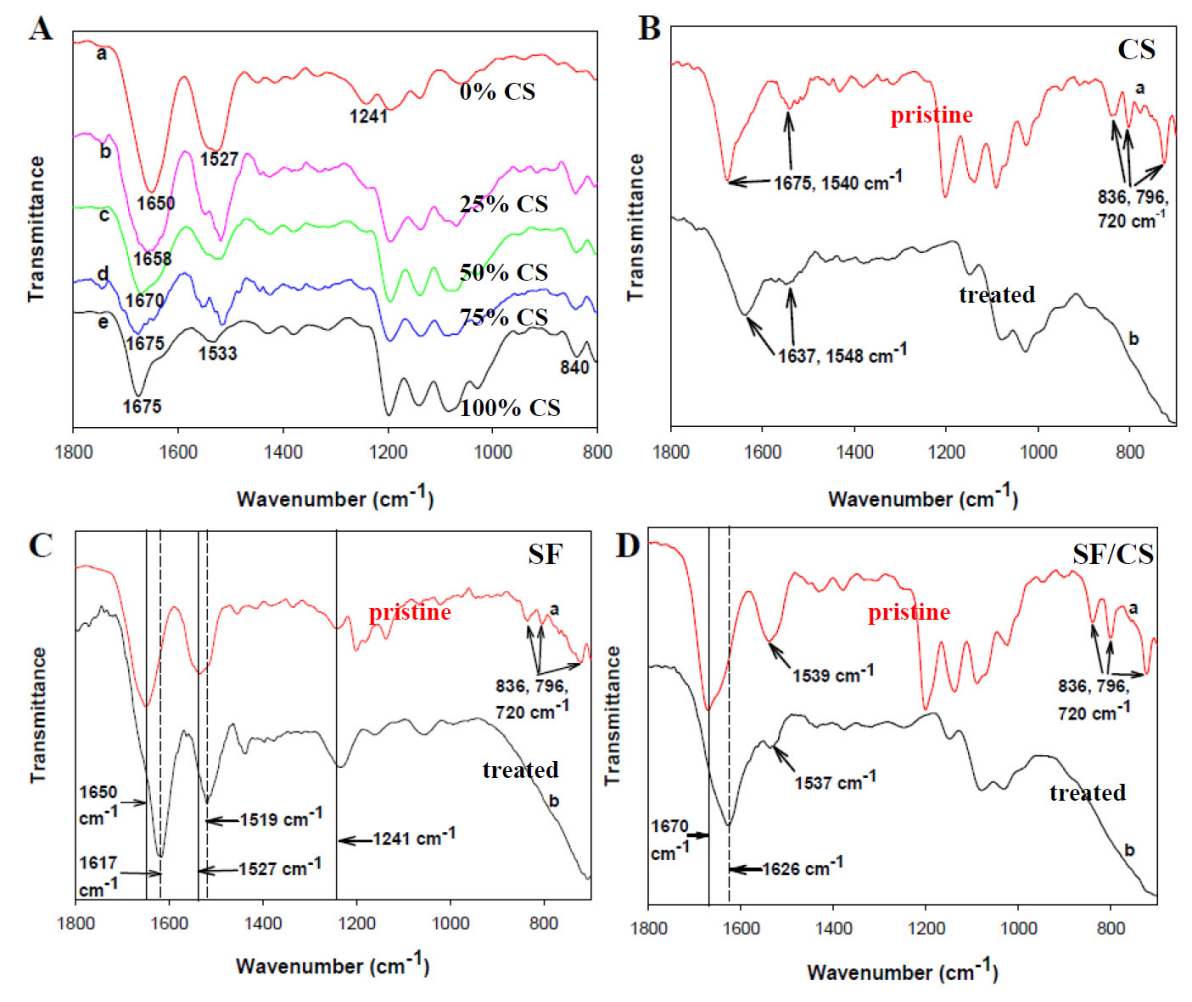
**FTIR spectra**. (**A**) FTIR spectra of pristine SF/CS NFs with (a) 0%, (b) 25%, (c) 50%, (d) 75%, and (e) 100% CS. FTIR spectra of pristine and chemically treated (**B**) CS, (**C**) SF, and (**D**) 50% SF/50% CS nanofibers (curve a is pristine and curve b is chemically treated).

The change of SF conformation from random coil to β-sheet structure and TFA removal were subject to FTIR studies. The characteristic transmittance peaks of pristine SF NFs were located at 1,650 (amide I), 1,527 (amide II), and 1,241 cm^-1 ^(amide III) [26,27]. The characteristic transmittance peaks of pristine CS NFs are protonated amino groups (-NH_3_^+^) around 1,675 and 1,533 cm^-1 ^[23]. With the increase of CS content in SF/CS composite NFs, the transmittance peak at 1,650 cm^-1 ^moves up to 1,658, 1,670, and 1,675 cm^-1 ^for 25%, 50%, and 75% CS, respectively (Figure 4A). The characteristic transmittance peaks of pristine CS NFs reveal TFA salt formation by three peaks from 720 to 840 cm^-1 ^together with amino group (-NH_3_^+^) around 1,675 and 1,540 cm^-1 ^(Figure 4B, curve a). The characteristic transmittance peaks of treated CS NFs are stretching of C = O and NH_2 _around 1,637 and 1,548 cm^-1^. However, the three transmittance peaks associated with TFA salts at 720, 796, and 836 cm^-1 ^disappeared after alkaline treatment (Figure 4B, curve b), which could be also observed from Figures 4C, D[22,23]. The characteristic transmittance peaks of pristine SF NFs are 1,650 (amide I) and 1,535 cm^-1 ^(amide II), which could be attributed to the random coil protein conformation. These peaks shift to 1,617 and 1,519 cm^-1 ^after ethanol treatment, indicating conformation structure change from random coil to β-sheet (Figure 4C) [26,27]. The characteristic transmittance peaks of pristine 50% SF/50% CS NFs show both SF and CS characteristic peaks (Figure 4D). Peak shifts at 1,670 and 1,539 cm^-1 ^again confirm the conformation change of SF in the composite NF after chemical treatment, and the C = O and NH_2 _stretching bands of CS merge with SF bands in the composite [28].

**Figure 5:**
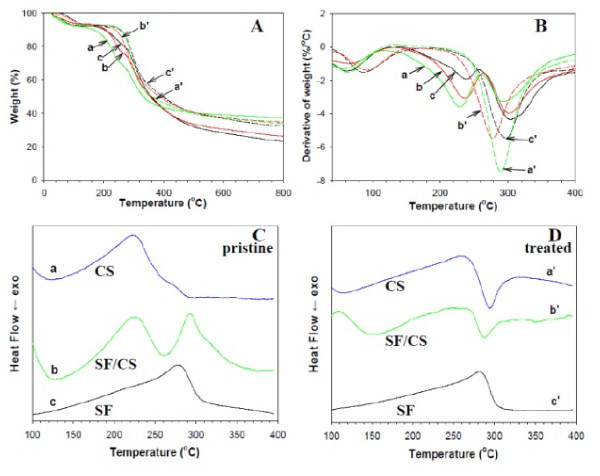
**TGA, DTG, and DSC analyses**. (**A**) TGA, (**B**) DTG, and (**C**, **D**) DSC analyses of pristine and chemically treated CS (a, a'), 50% SF/50% CS (b, b'), and SF (c, c') nanofibers. Curves a, b, and c are pristine nanofibers; curves a', b', and c' are chemically treated nanofibers.

Two decomposition peak temperatures are observed for pristine SF/CS NFs in contrast to only one for treated NFs from TG (Figure 5A) and differential thermogravimetry [DTG] (Figure 5B) analysis [28,29]. The first peak corresponds to the decomposition of the salt NH_3_^+^-CF_3_COO^- ^while the second peak is due to the decomposition of SF and CS. After chemical treatment, the peak temperature due to salts formed from TFA and protonated amines at 235°C disappeared, and only the second peak at approximately 290°C could be observed, reconfirming the complete removal of TFA by binding with ammonium ions during the chemical treatment process [24].

DSC analysis reveals unique features for pristine (Figure 5C) and treated (Figure 5D) NFs of different CS contents. In a recent study, a strong exothermic peak at 305°C and a strong endothermic peak at 280°C were reported for pure CS and SF, respectively [28,29]. Compared with DTG, DSC analysis shows that only the NH_3_^+^-CF_3_COO^- ^salt in CS leads to a new peak with the disappearance of the exothermic peak at 305°C for CS and appearance of a new endothermic peak at 222°C (Figure 5C). For pristine SF/CS composite NFs, two endothermic peaks due to SF and TFA-CS appear (Figure 5C). After adding ammonia, the endothermic peak of TFA-CS at 222°C changed back to the strong CS exothermic peak at 298°C by completely removing TFA from NFs with ammonium ions (Figure 5D). Also, the strong endothermic peak of SF located at 278°C shifts to a higher temperature at 282°C after the protein structure being transformed into the β-sheet crystallization by ethanol (Figure 5D) [30]. Interestingly, the DSC curve of treated 50% SF/50% CS composite NFs shows a strong exothermic peak similar to that of CS instead of the endothermic peak associated with pure SF (Figure 5D).

**Figure 6:**
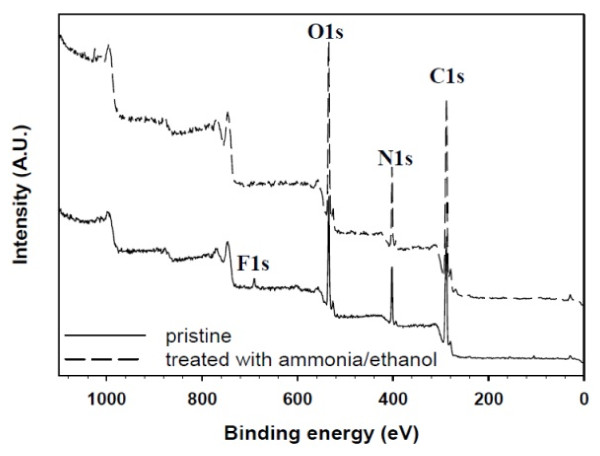
**XPS survey scan spectra of 50% SF/50% CS nanofibers' surfaces before and after chemical treatment**.

To ascertain further the complete removal of TFA, the surface chemical composition of SF/CS NFs was compared by XPS analysis in Figure 6 which shows the survey scan spectra of pristine and treated SF/CS NFs. As can be seen from Figure 6, all XPS spectra have three separated peaks which correspond to C1s (285 eV), N1s (403 eV), and O1s (532 eV). However, only a distinct F1s peak at 691 eV (approximately 1.11%) exists in the spectrum of pristine NF, but not in that of the treated NF as residual TFA was successfully removed from the fiber surface.

**Figure 7:**
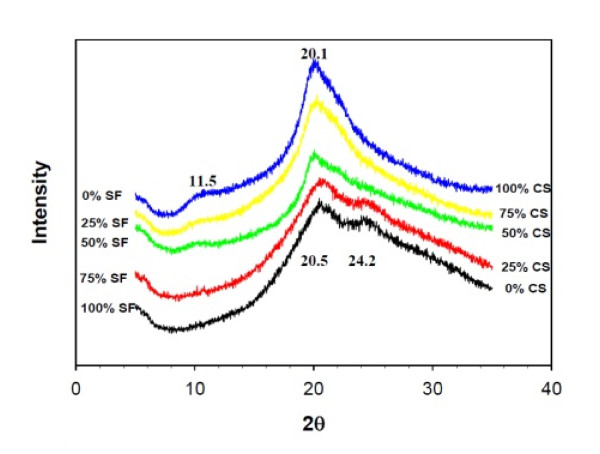
**XRD patterns of treated SF/CS nanofibers with different CS content**.

To elucidate the crystalline structure of treated SF/CS composite NFs, results of XRD analysis are shown in Figure 7, which reveal four distinct peaks in the region between 5° and 35°. The diffraction peak at 20.5° denotes the random coil conformation, and 24.2° denotes the β-sheet structure of SF [31]. For CS, two diffraction peaks at 11.5° and 20.1° could be identified, which are characteristics of the hydrated crystalline structure of CS. The SF/CS composite NF shows diffraction peaks from both CS and SF. However, CS seems to dominate over SF in determining the crystalline structure of the composite NF as diffraction peaks associated with SF are only visible with ≤ 25% CS content. Segregation of CS and SF will therefore not happen in the NF as XRD results indicate the presence of one component will influence the crystalline structure of the other through molecular interactions, which is consistent with the distribution of each component in the NF from confocal images in Figure 2.

### Culture of hFOB on SF/CS composite nanofibers

**Figure 8:**
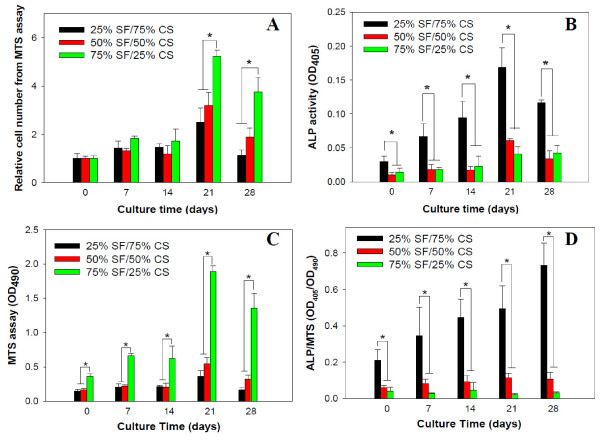
**Proliferation and differentiation of hFOB cultured on SF/CS nanofibers with different CS contents (n = 5)**. (**A**) Relative cell number determined by MTS assays with cell number at day 0 taken as one for each group, (**B**) alkaline phosphatase [ALP] activities, (**C**) absolute cell number determined by MTS assays, and (**D**) ALP activities normalized to cell number. *p < 0.05.

Figure 8 shows cell proliferation by MTS assays and cell differentiation by ALP activities of hFOB. For relative cell number, the number of viable cells at day 0 after cell attachment for 4 h was taken as one for each sample to compare the proliferation rate of cells in different NF scaffolds. The cell number and ALP activity increased with culture time but show clear dependence on the composition of the NF. The SF/CS NF with the least amount of CS (25%) significantly promotes (*p *< 0.05) cell proliferation compared to other groups after day 21 (Figure 8A). The absolute cell number for hFOB cultured on the same NF also showed significant difference (*p *< 0.05) compared to other groups throughout the culture period (Figure 8C). On the contrary, the osteogenic differentiation of hFOB shown from the bone-specific ALP activity (total activity or normalized to cell number), which is essential for the process of bone mineralization, is significantly enhanced (*p *< 0.05) on the NF containing the highest amount of CS (75%) throughout the culture period (Figures 8B, D). Therefore, hFOB responds to SF and CS in the NF differently, with the former providing a cue for cell proliferation while the latter for osteogenic differentiation. In a recent study, osteoblasts were found not to be able to proliferate on CS NFs [32]. On the contrary, mesenchymal stem cells attached to calcium phosphate-CS scaffolds successfully differentiated down the osteogenic lineage and expressed elevated levels of ALP [33].

**Figure 9:**
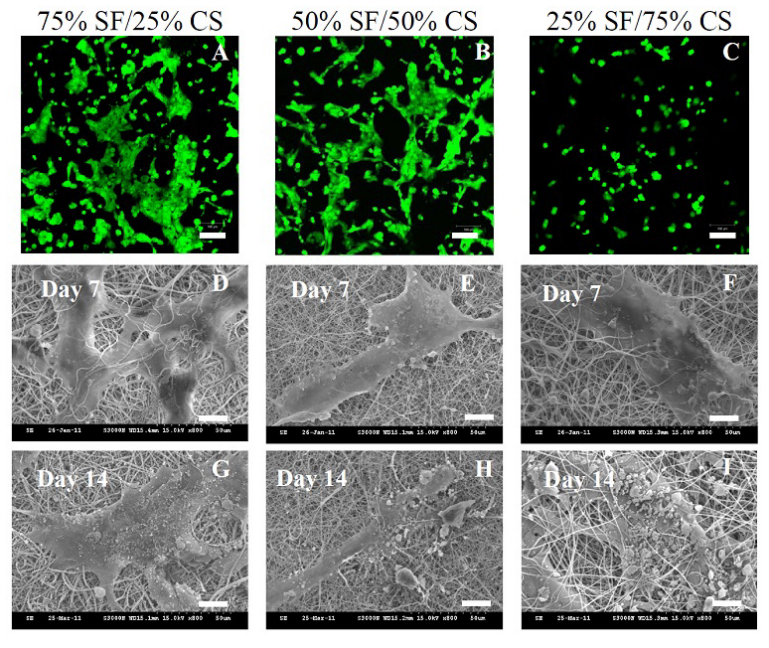
**Live/dead cell staining and SEM images of hFOB**. (**A **to **C**) Live (green)/dead (red) cell staining of hFOB after 1 day cell culture (bar = 100 μm) and SEM images of hFOB after 7 days (**D **to **F**) and 14 days (**G **to **I**) cell culture on SF/CS nanofibers with different CS contents (bar = 20 μm).

The distributions and viability of hFOB in NFs were observed using confocal microscopy after live/dead cell staining. After 1 day in culture, all cells were live (stained green) in all NFs, and dead cells (stained red) are not observed (Figure 9). The difference in number of live cells and spreading of cells observed in the figure is due to the composition of the NF. More live cells could be observed in 25% CS NFs, and the cells spread more than those in 75% CS NFs and distribute more evenly. As SF could promote more cell attachment and growth than CS from Figure 8, it is apparent that SF also promotes cell spreading and lead to flat morphology of cells compared to CS which leads to a round-cell morphology. Figure 9 also shows the SEM images of cell-scaffold constructs after 7 and 14 days of culture where the morphology of osteoblasts and the mineral particles secreted by osteoblasts are observed. That CS could promote mineralization of hFOBs with elevated ALP secretion as shown in Figure 8D is confirmed as cells in 25% SF/75% CS NFs can secrete the highest amount of mineral particles from SEM observations.

## Conclusions

Composite SF/CS NFs covering the whole range of CS content can be prepared by electrospinning. Post-treatment with ammonia/ethanol solution can totally eliminate harmful TFA residue from the NF and render it insoluble in cell culture medium and useful as tissue engineering scaffold. hFOB cells could proliferate and differentiate on the NFs. Each component in the composite NF shows distinct effect on cell behavior, with SF promoting cell proliferation and CS enhancing osteogenic differentiation. By choosing a suitable blend of each component in the composite, 50% SF/50% CS NFs will be a promising candidate scaffold for bone tissue engineering.

## Competing interests

The authors declare that they have no competing interests.

## Authors' contributions

J-PC and S-HC designed the experiment. S-HC prepared and analyzed the samples. S-HC and G-JL carried out cell culture experiments. All authors read and approve the final manuscript.
